# High-Throughput IgG Epitope Mapping of Tetanus Neurotoxin: Implications for Immunotherapy and Vaccine Design

**DOI:** 10.3390/toxins15040239

**Published:** 2023-03-24

**Authors:** Salvatore G. De-Simone, Paloma Napoleão-Pêgo, Guilherme C. Lechuga, João P. R. S. Carvalho, Larissa R. Gomes, Sergian V. Cardozo, Carlos M. Morel, David W. Provance, Flavio R. da Silva

**Affiliations:** 1 Center for Technological Development in Health (CDTS)/National Institute of Science and Technology for Innovation in Diseases of Neglected Populations (INCT-IDPN), Oswaldo Cruz Foundation (FIOCRUZ), Rio de Janeiro 21040-900, RJ, Brazil; pegopn@gmail.com (P.N.-P.); gclechuga@gmail.com (G.C.L.); larissagomesr@gmail.com (L.R.G.);; 2 Laboratory of Epidemiology and Molecular Systematics (LESM), Oswaldo Cruz Institute, Oswaldo Cruz Foundation (FIOCRUZ), Rio de Janeiro 21040-900, RJ, Brazil; 3 Post-Graduation Program in Science and Biotechnology, Department of Molecular and Cellular Biology, Biology Institute, Federal Fluminense University, Niterói 22040-036, RJ, Brazil; 4 Department of Health, Graduate Program in Translational Biomedicine (BIOTRANS), University of Grande Rio (UNIGRANRIO), Caxias 25071-202, RJ, Brazil; sergian.cardozo@unigranrio.edu.br

**Keywords:** tetanus neurotoxin, B-cell linear epitopes, immunological diagnostic, peptide ELISA

## Abstract

Tetanus is an acute, fatal disease caused by exotoxins released from *Clostridium tetani* during infections. A protective humoral immune response can be induced by vaccinations with pediatric and booster combinatorial vaccines that contain inactivated tetanus neurotoxin (TeNT) as a major antigen. Although some epitopes in TeNT have been described using various approaches, a comprehensive list of its antigenic determinants that are involved with immunity has not been elucidated. To this end, a high-resolution analysis of the linear B-cell epitopes in TeNT was performed using antibodies generated in vaccinated children. Two hundred sixty-four peptides that cover the entire coding sequence of the TeNT protein were prepared in situ on a cellulose membrane through SPOT synthesis and probed with sera from children vaccinated (ChVS) with a triple DTP-vaccine to map continuous B-cell epitopes, which were further characterized and validated using immunoassays. Forty-four IgG epitopes were identified. Four (TT-215-218) were chemically synthesized as multiple antigen peptides (MAPs) and used in peptide ELISAs to screen post-pandemic DTP vaccinations. The assay displayed a high performance with high sensitivity (99.99%) and specificity (100%). The complete map of linear IgG epitopes induced by vaccination with inactivated TeNT highlights three key epitopes involved in the efficacy of the vaccine. Antibodies against epitope TT-8/G can block enzymatic activity, and those against epitopes TT-41/G and TT-43/G can interfere with TeNT binding to neuronal cell receptors. We further show that four of the epitopes identified can be employed in peptide ELISAs to assess vaccine coverage. Overall, the data suggest a set of select epitopes to engineer new, directed vaccines.

## 1. Introduction

Tetanus is a highly fatal, noncommunicable, and preventable disease caused by infections with *Clostridium tetani* [1,2,3]. Children, women, and newborns are at the highest risk of acquiring tetanus due to low immunization rates and unhygienic deliveries [4]. Consequently, tetanus is a public health problem with a recorded worldwide yearly incidence of a million neonatal cases and 34,000 deaths in 2015, according to WHO estimates [4]. The child mortality rate ranges from 20 to 50% [5,6,7,8]. The vaccination schedule of the Brazilian national immunization program advocates for an initial dose of the tetanus vaccine during the first two months of life with two more doses at 4 months and at 6 months of age. In addition, two boosters are administered: one at 15 months of age and the other at 4 years old. Children who complete the vaccination cycle show a high level of protection, avoiding death. Even the immunization of pregnant women, or women of childbearing age, reduces neonatal tetanus mortality by about 94% [4,7].

Virulence is associated with tetanus neurotoxin (TeNT), a 150 kDa AB toxin excreted by the bacteria consisting of a light chain (Lc) of 50 kDa and a heavy chain (Hc) of 100 kDa that are joined by a disulfide bond [9]. Lethality is related to a block in neurotransmitter release caused by the N-terminal zinc-dependent metalloendopeptidase in the Lc and its cleavage of VAMP/synatobrevin, a membrane-associated protein involved in synaptic vesicle fusion [10].

Residues 233 to 237 in the N-terminus of the Lc present a His-Glu-XX-His Zn^2+^ motif that forms a primary sphere of residues in the enzymatic active site for coordinating the binding of a zinc ion [11]. A secondary sphere of residues determines the proteolytic specificity of the peptidase activity [10]. The Hc is responsible for entry of the toxin into the cytoplasm of neurons and consists of two domains [12]. A translocation domain is located in the N-terminal region, which is responsible for internalization and translocation into the neuronal cytosol through a clathrin-mediated pathway involving dynamin and AP-2 [13]. The receptor-binding domain resides in the C-terminal region that forms a double bond to membrane gangliosides [1,14,15]. The C-terminal domain, often referred to as the C-fragment, can be further divided into two subdomains of 25 kDa, the N-proximal subdomain (HcRN) and the carboxy-terminal subdomain (HcRC) [16]. In the HcRC subdomain, there is a crystalline structure that is primarily responsible for binding gangliosides [17]. Gangliosides are a type of glycosphingolipid in the plasma membrane composed of sialic acid residues with an acidic nature that can serve as signaling receptors between cells and the extracellular environment [18]. Although gangliosides are present in all tissues, they are most abundant in nerve cell membranes [19]. With respect to TeNT, gangliosides have a high affinity for both C-fragment binding sites and can be considered as dual-function receptors [14]. 

Despite extensive vaccination, tetanus remains an significant cause of death worldwide, particularly in developing countries [20,21]. Vaccines against tetanus are clinically effective [22,23], rarely present side effects [24], and are composed of a chemically inactivated tetanus toxoid (CITTo). However, CITTo is a crude preparation that can contain hundreds of *Clostridium tetani* proteins, and the biomedically relevant component is present at variable levels that sometimes only represent a minor percentage of vaccine mass [25]. Furthermore, the toxoids do not resemble the native toxins after formaldehyde treatment in vitro and apparently lack some important neutralizing epitopes. As a result, these modified toxoids may only elicit mild or moderate protection in vivo [26], which necessitates multiple injections of a vaccine to reach a long-lasting immunization capable of protecting against tetanus. This can explain the ineffectiveness of vaccination programs in some developing countries, which can be exacerbated by low vaccination coverage [27,28].

Together with other issues, such as the use of formaldehyde, which complicates manufacturing procedures through production risks and pollution, many countries are actively researching for the development of a new generation of safer and more effective tetanus vaccines through biotechnological approaches that are capable of lifelong immunization against tetanus [29]. A good protective immune response against TeNT after parenteral immunization has been demonstrated using the HcR domain, which encompasses the host receptor-binding motif [13,26,30]. Recently, a recombinant, non-tagged isoform of the Hc domain of the TeNT alone was successfully evaluated in mice [31]. A different recombinant tetanus vaccine was engineered with eight individual amino acid mutations (8MTT) in TeNT to genetically inactivate catalysis, translocation, and host receptor-binding functions. This protein retained 99.4% amino acid identity to native tetanus toxin (TT) and elicited a potent immune response in mice, which displayed an effective vaccine potency against a TT challenge [32]. 

Another approach is to generate a highly directed vaccine focused on sites within the TTo antigen that is directly involved with the protective humoral immune response observed with vaccination. However, a structurally resolved analysis of the epitopes in TeNT underlying the molecular mechanisms of antibody neutralization is unavailable. Eleven different epitopes have been deposited in the IEDB (Immune Epitope Database and Analysis Resource; https://www.iedb.org/home_v3.php (accessed on 15 January 2023)) database, and several works have described the isolation of several monoclonal antibodies without defining their cognate binding site [33,34,35,36,37,38,39,40,41]. Yet, the structure of these epitopes is not known and many of the epitopes still needed to be identified. To reveal the full range of epitopes recognized in TeNT that are elicited by current tetanus vaccines, a complete and careful immunological mapping was performed using the high-resolution methodology of printable SPOT synthesis. The epitopes identified along with their implication in immunotherapy and vaccination are discussed. In addition, a set of four epitopes were employed in peptide ELISAs to evaluate the coverage of vaccinations post-pandemic in a group of children living in Rio de Janeiro, Brazil.

## 2. Results

### 2.1. Identification of the Immunodominant IgG Epitopes in TeNT 

**Figure 1:**
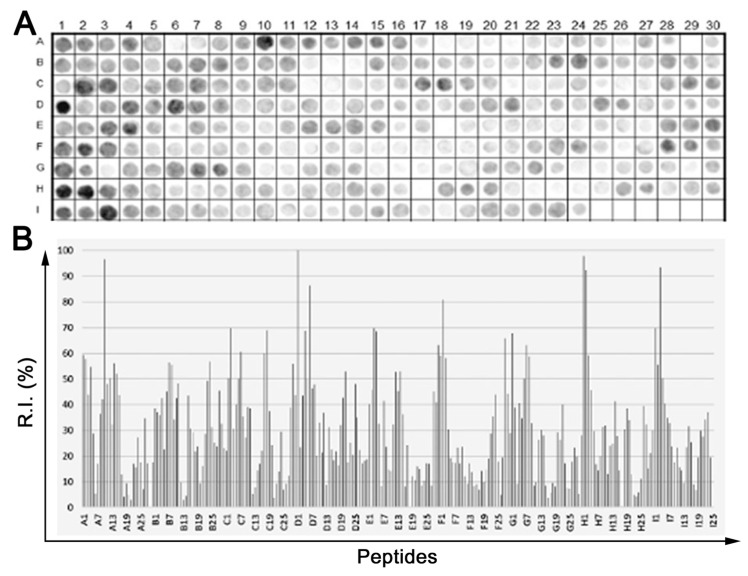
Mapping of DTP vaccine induced IgG epitopes within TeNT. A library of 264, 15-mer peptides (listed in Table S1) with a 10 residue overlap and covering the coding sequence of TeNT (P04958; 1315aa) was prepared using SPOT synthesis. (**A**) Image of the chemiluminescence signal from the library after an incubation with a pooled of sera from children vaccinated with DTP (*n* = 15) followed by labeling with a goat anti-human IgG alkaline phosphatase-labeled secondary antibody. (**B**) Graph of the signal intensity normalized to 100% with the positive control and 0% with the negative control. Epitopes were identified within consecutive reactive peptides (Table 1).

Linear epitopes often encompass 4 to 9 amino acid residues in size, which suggests that a detectable signal of high confidence can be observed across a library of 15-mer peptides overlapping by 10 amino acids and containing epitope motifs. Applying this principle, signal intensities for each of the peptides were mapped back to the respective TeNT sequence from which the peptides were derived. By plotting signal intensity as a function of peptide position in the toxin sequence, the binding profile of the TeNT pair was obtained. In Figure 1, panels A and B present the role of each peptide and the measured reactivity intensity, respectively, from the chemiluminescent detection of IgG antibodies in sera pooled from vaccinated children (*n* = 15). The absolute signals were normalized to percentages using 100% as defined by the positive control. The list of the synthetic peptides’ positions on the membranes can be found in Table S1. Fourth-three IgG epitopes were identified in TeNT with 14 epitopes in the L-chain and 29 epitopes in the H-chain (Table 1).

### 2.2. Epitope Reactivity by ELISA-MAP4

**Figure 2:**
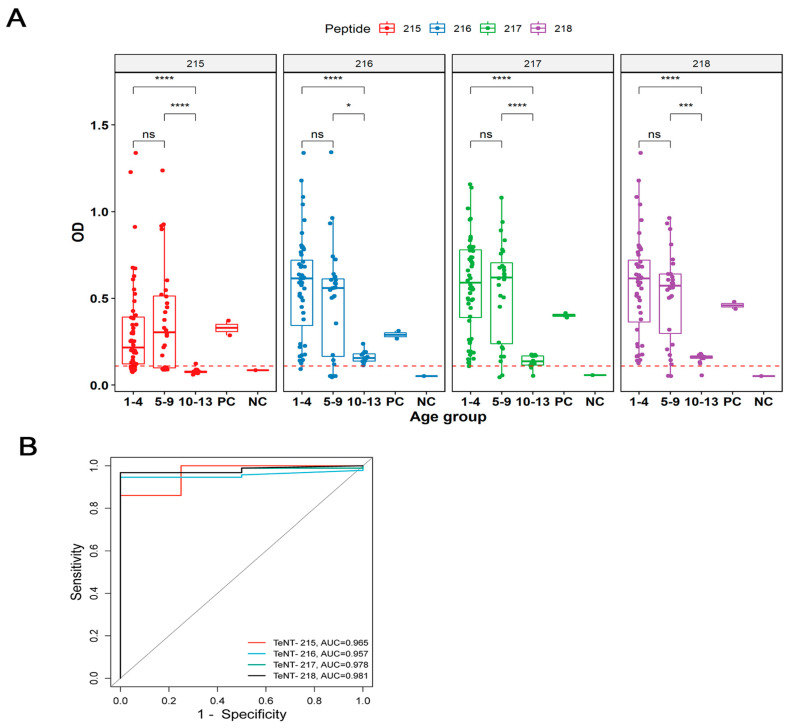
Reactivity of serum from DTP vaccinated children against TeNT-MAP4 by an in-house ELISA. (**A**) Individual sera (*n* = 92) were tested through peptide ELISAs that utilized either Peptide 215 (MAP4-VPERYEFGTKPEDFN), 216 (MAP4-EYVPTFDNVIENTTS), 217 (MAP4-EKTLNDYKFQ FDSNG), or 218 (MAP4-GTVNTQFQYEYKIYS). The dashed lines represent the calculated cut-off values of each assay. The ROC analysis (**B**) showed that the sensitivity of the in-house peptide ELISAs were 76% for TeNT-215, 100% for TeNT-216, 100% for TeNT-217, and 100% for TeNT-218 (100%). All MAPs displayed 100% specificity except TeNT-218 (71%). Statistically significant (* *p* < 0.05; *** *p* < 0.001; **** *p* < 0.0001) and ns: not significant.

The method of synthesizing multiple antigenic peptides (MAP) was applied to improve the immunoreactivity of synthetic peptides that represent distinct regions of TeNT. Four selected tetrameric MAP with multiple incorporated overlapping B-cell epitopes were combined with a particular TeNT sequence representing the peptide in the C-terminus of the TeNT and evaluated with a panel of sera from children who sought vaccination in 2021 at a clinic of the Brazilian Ministry of Health. Sera were distributed by age, and the diagnostic performance of peptides [215 (MAP4-VPERYEFGTKPEDFN), 216 (MAP4-EYVPTFDNVIENTTS), 217 (MAP4-EKTLNDYKFQFDSNG) and 218 (MAP4-GTVNTQFQ YEYKIYS)] was evaluated with sera from ninety-three DTP vaccinated children (1–4 years, 5–9 years, 10–13 years) who were tested on an ELISA peptide-based assay (Figure 2)**.**

All ChVS were collected at the same time. Children aged 1–4 years old had received the three doses of the vaccine according to the vaccination schedule of the Brazilian Ministry of Health. Children aged 5–9 years old had received the two booster applications at 15 months and 4 years in addition to the three doses of vaccines. Children aged 10–13 years had completed the full vaccination cycle and were also vaccinated with the childhood DT (diphtheria and tetanus) vaccine administered at over 7 years of age. The main observed difference in the age groups was that the sera from 10–13-year-olds showed the lowest titer, which may have been due to not having completed the vaccination cycle for childhood DT vaccine (booster) necessary to maintain the level of circulating IgG antibodies. Even all adults are advised to receive the adult DT vaccine every 10 years to support the degree of protection.

The performances of the peptides were compared to a commercial kit for classic tetanus IgG (Serion Brazil, Pinhais, PR, Brazil). A significant number of the 92 DTP-positive ChVS reacted with the highly conserved 215–218 peptides. Standard negative human sera from a commercial kit were used in peptide ELISA for peptides 215–218 but did not react. Negative controls were used to determine the cut-off (0.109). Peptide ELISAs using the 215–218 antigens demonstrated that no significant statistical difference was found between ChVS from 1–4- and 5–9-year-olds. However, the group of 10–13-year-olds presented a statistically significant decline in reactivity with a mean optic density reduction that varied from 66 to 79%. The tests presented different performances. Peptide TT-215 showed a greater number of negative reactions (*n* = 32), followed by peptide TT-216 and 217 (*n* = 5) and TT-218 (*n* = 3). The maximum accuracy value is one, which represents a perfect test. Our peptide in-house ELISA immunoassay presented an area under the curve that varied from 0.95 to 0.98 (Figure 2B). 

**Figure 3:**
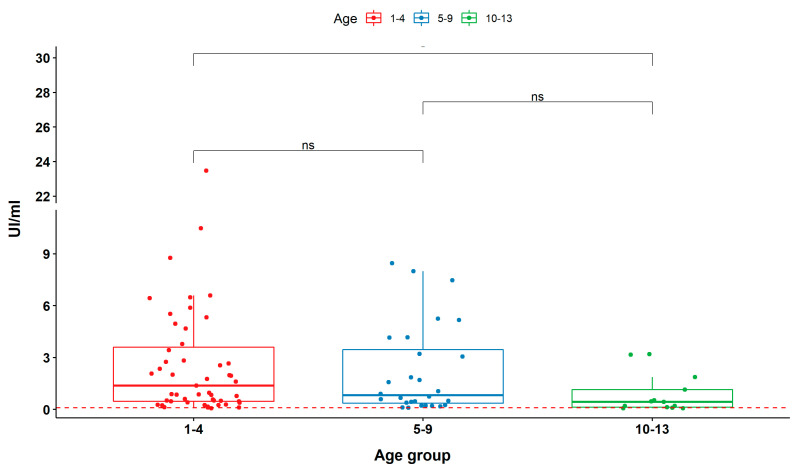
Reactivity of sera from vaccinated children (ChVS, *n* = 93), distributed into three different age groups, evaluated using a commercial ELISA kit. The activity of IgG antibodies is expressed in international units per mL (UI/mL). Cut-off (<0.1 UI/mL; red dashed line) was based on manufacturer’s manual according to standard curve and four-parameter logistic (4PL) regression.

The same samples of ChVS were also analyzed using a commercial kit. According to the manufacturer, the qualitative ELISA has >99% sensibility and 97% specificity to monitor the immunological state of vaccinated individuals. Applying the cut-off (0.1 UI/mL), the tests were nearly equivalent to that of the SERION ELISA classic tetanus IgG. Of 93 ChVS, 88 were confirmed as positive and 5 were negative (<0.1 UI/mL), indicating insufficient immunological protection and the need of a booster dose (Figure 3). The concordance rate between the peptides tested and commercial kit ELISA was 77.5, 100, 100, and 97.9 % for TT-215, TT-216, TT-217, and TT-218, respectively. 

### 2.3. The Spatial Location of TeNT Reactive Epitopes 

**Figure 4:**
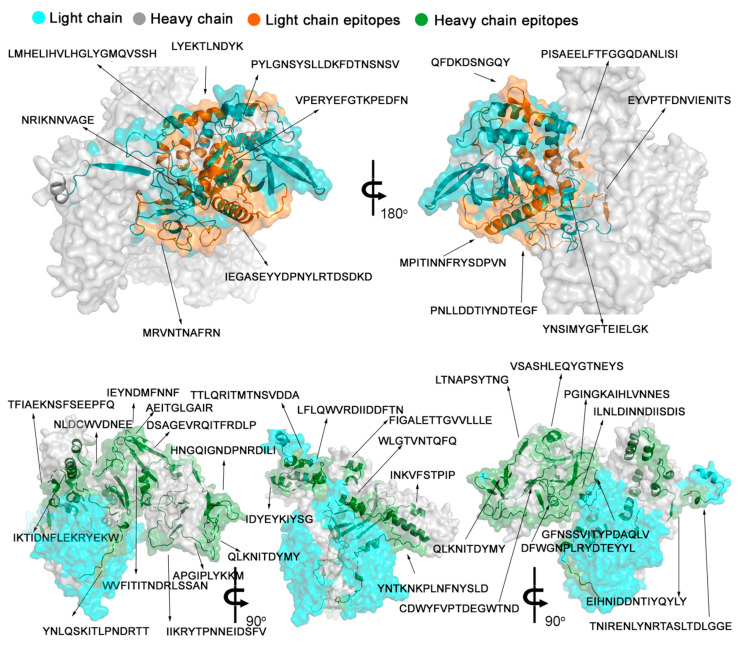
Three-dimensional structure of TeNT (A) with the position of the 43 IgG epitopes identified through SPOT synthesis. Molecular model overlay of neutralizing epitopes within the TeNT protein. The image was constructed using PyMol based on the crystal structure of the neurotoxin (PDB: 1xdt). The catalytic (cyan), translocation (green), and receptor-binding (yellow) domains are depicted in cyan, green and yellow, respectively, with the epitopes in magenta.

**Figure 5:**
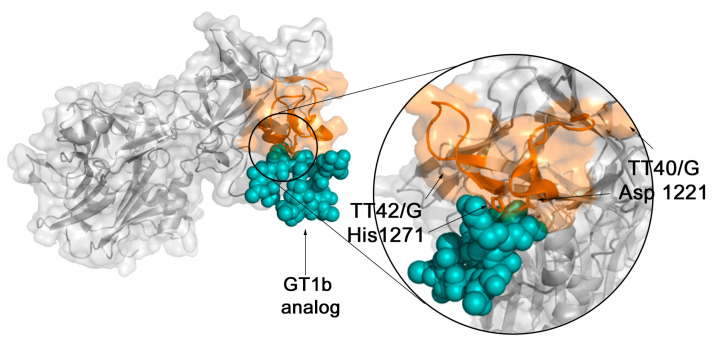
The Hc fragment of tetanus toxin interacting with ganglioside GT1b analog (PDB: 1fv3). Identified epitopes TT-41/G and TT-43/G (orange) show close contact with ganglioside receptor analog of GT1b (N-acetyl-alpha-neuraminic acid-(2-3)-beta-D-galactopyranose-(1-3)-2-acetamido-2-deoxy-beta-D-galactopyranose-(1-4)-[N-acetyl-alpha-neuraminic acid-(2-8)-N-acetyl-beta-neurami nic acid-(2-3)]beta-D-galactopyranose-(1-4)-beta-D-glucopyranose) interacting with Asp 1221 and His 1271 residues.

The spatial localizations of the epitopes within the structure of TeNT were mapped onto the crystallographic model [42,43] available in PDB (PDB: 1xdt) (Figure 4), which displays the spatial location of the forty-three reactive epitopes identified through the SPOT synthesis array experiments (Table 1). Thirteen of the identified epitopes were exclusively in loop/coil structures, while eleven shared amino acids in coil and helix or β-sheet (Table 1 and Figure 4). However, all were present on the protein surface and accessible to the solvent (data not shown). Figure 5 depicts the HcR domain with the neutralizing epitopes TT-41/G and TT-43/G. 

### 2.4. Validation of TeNT IgG Epitopes Deposited in the IEDB Database

**Figure 6:**
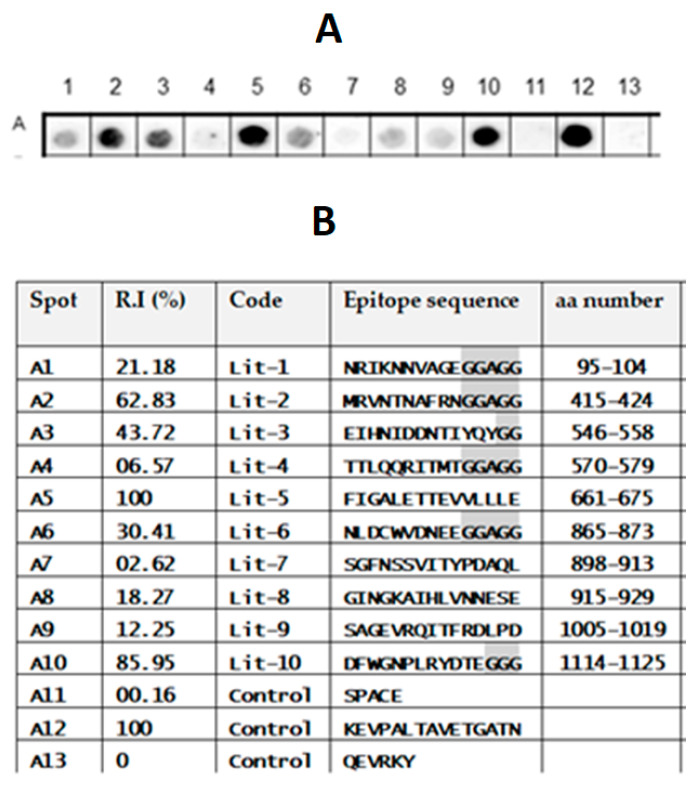
List of the TeNT IEDB-deposited B-cell linear epitopes reanalyzed using SPOT synthesis. (**A**) Image of peptide reactivity using the same pool (*n* = 15) of ChVS used in the previous analysis. (**B**) The ten-epitope peptides were ordered with 15 residues, and those with fewer residues were synthesized with the addition of two Gly or GGAGG residues (shaded in gray) in the C-terminus until the complete 15 depositions were completed.

A list of TeNT B-linear epitopes is described in the IEDB database (Figure 6B). However, some of the epitopes were not identified in our SPOT synthesis analysis screening. Therefore, an experiment was designed wherein only the 10 peptides described in the IEDB were synthesized and analyzed according to the analysis in Figure 1. Initially, an antibody membrane exposure time of 2 min was used and a cutoff of 30% normalized signal intensity was maintained to define reactivity. The results were not encouraging, since nine of the epitopes were not identified (Supplementary Figure S1). By extending the exposure time to 5 min, nine of the ten peptides were identified as epitopes (Figure 6A,B), except for the Lit-7 peptide (SGFNSSVITYPDAQL), which remained undetectable. Overall, these studies indicated that four of the nine peptides listed in the IEDB and detected with 5 min of exposure are minor IgG epitopes (Figure 6A,B).

### 2.5. Identification of Subclass of Immunoglobulins through ELISA

**Figure 7:**
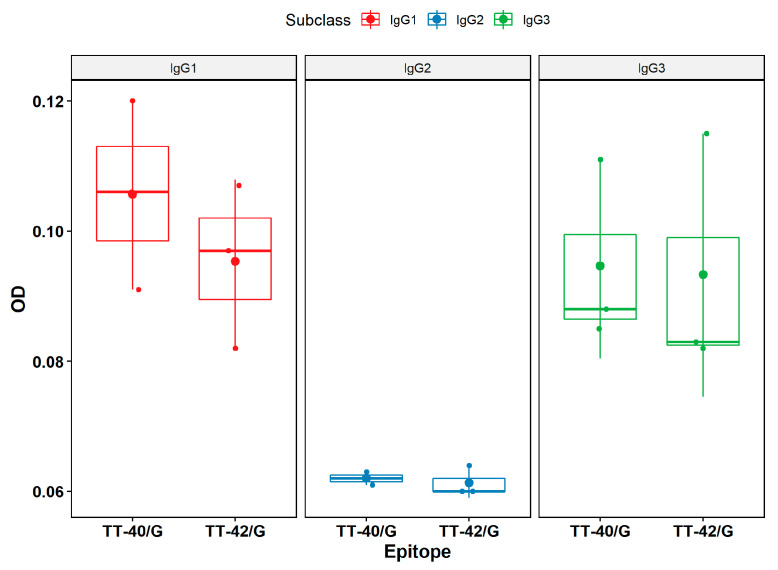
Identification of the subclasses of immunoglobulins that bind to TT-41/G (YPKDGNAFN NLDRIL) and TT-43/G (HNGQIGNDPNRDGGG) peptides through ELISA.

To determine which subclasses of human IgG are induced for the three main neutralizing epitopes recognized in this study, TT-8/G (SAEELFTFGGQDAGG), TT-41/G (YPK DGNAFNNLDRIL), and TT-43/G (HNGQIGNDPNRDGGG) peptides were synthesizede as unique peptides and analyzed through ELISA using subclass-specific secondary antibodies (Figure 7). The TT-8/G epitope induces IgG_1_ antibodies (data not shown), and the TT-41/G and TT-43/G epitopes IgG1 and IgG_3_ antibodies, respectively.

### 2.6. Cross-Immune IgG Epitopes 

To investigate whether the TeNT epitopes could present cross-immunity with other proteins, the Pir program (https://proteininformationresource.org/; 10 October 2022) was used to analyze the peptide sequences with homology searches (similarity with four or more consecutive amino acids) with possible sequences deposited in the bank. The results showed that all of the epitopes were unique to *Clostridium tetani,* indicating that immunity to TeNT is species-specific.

## 3. Discussion

The high-throughput, immune-profiling peptide array synthesized directly onto cellulose membranes allowed the identification of many antigenic determinants for the TeNT recognized by sera of children vaccinated with a single dose of the DTP. Forty-three epitopes covering the full extent of the bacterial toxin were identified (Table 1). This neurotoxin is secreted by Corynebacterium and prevents the release of inhibitors of synapse function, leading to continued neuromuscular activation and spastic paralysis [44,45,46,47].

Fifteen of the identified epitopes (TT-1/G to TT-15/G) were positioned in the enzymatic L-chain peptide (aa1–aa500), although no epitope covered Glu-271 and Tyr-375, which are essential amino acids in the catalytic domain [9]. Since the spatial folding of the protein plays a decisive role in enzymatic activity, the presence of two epitopes (TT-6/G and TT-7/G) around the active site could exercise an important steric effect when bound by an antibody that should effectively block or diminish enzymatic activity. In the H-chain, which is responsible for the cytosolic translocation of the toxin, 28 epitopes were identified, with 15 in the HcT membrane-translocation domain (aa501–aa1000) and 13 in the heavy domain (HcR) that is involved with toxin binding to nerve terminals within neuromuscular junctions (aa 1001–1500). 

The structure of the Hc domain is well known [48]. Louch et al. [49] showed that the amino acids Asp-1221, His-1270, and Try-1288 are essential residues for binding to GT1b by the tetanus toxin HcR fragment using site-directed mutagenesis. Our work demonstrated that at least two of these amino acids (Asp-1221 and His-1270) are components of the epitopes TT-41/G and TT-43/G, suggesting that these sequences are responsible for the production of antibodies that can sterically interfere with interacting with neuronal cells.

The tetanus vaccine has been used since 1924 [50], and still the current production process for TeNT vaccines continues to be largely based on the traditional methods first used to detoxify TeNT [51]. Recently, Metz et al. [52] revealed that the chemical modifications used for detoxification of TeNT during vaccine production typically result in a combination of intramolecular crosslinks and formaldehyde-glycine attachments. In our study, the detoxification process apparently does not interfere in the presentation of linear B-cell epitopes in areas important for neutralizing TeNT, since the antibodies in the serum of DTP vaccinated children identified these regions. Furthermore, in a synthetic model, we have shown that all the forty-three B-linear epitopes identified are positioned on the molecule’s surface and, thus, are exposed to the immune system. Therefore, based on the function of the three domains, the 43 identified epitopes can present important functions such as the blocking or steric impairment of enzymatic activity and/or intracellular translocation and blockade of TeNT neuronal receptor-binding (HcR). Unfortunately, to our knowledge, there are only a few studies concerning the identification of key TeNT epitopes responsible for vaccine protection.

The LH chain (aa 2–457) of TeNT is a zinc-dependent metalloprotease, and its active site is composed of a primary sphere of residues coordinating the zinc atom and a secondary sphere of residues that determine the proteolytic activity and specificity. Residues Glu-271 and Tyr-375 are essential for proteolytic activity [9]. Looking at the epitopes identified (Table 1), we could discern that the amino acid Glu-271 occupies a central position in the TT-8/G epitope (269-AEELFTFGGQD-279), thus preventing it from exercising its enzymatic activity.

The tetanus toxin fragment C (TTFc) has been extensively studied both as a neuroprotective agent for central nervous system disorders owing to its neuronal properties [53], vaccination [54], and as a carrier protein for vaccines [55]. Indeed, it is derived from a part of the TeNT and, as such, retains its immunogenic properties without being toxic. Moreover, this fragment has been well characterized, and its entire structure is known. Our study identified that the epitopes TT-40/G (1211-YPKDGNAFNNLDRIL-1225) and TT-42/G (1271-HNGQIGNDPNRD-1282) are located in the RcH chain of the TeNT and are closely associated with the ganglioside receptor analog of GT1b through its interaction with Asp-1221 and His-1271 residues [56]. As expected, vaccination induced mainly IgG1 and IgG3 antibody subclasses for the three key peptides epitopes analyzed (TT-8, TT-41 and TT-43) (Figure 7), which is consistent with IgG being the most predominant immunoglobulin and the association of IgG3 with primary vaccination with tetanus toxoid [57].

Our SPOT synthesis analysis identified only a small set of epitopes predicted to contain single alpha helices and/or ß-sheet positioned amino acids, which is not usual for B-cell linear epitopes. It is possible that this discrepancy in the structure of the epitopes that do not present only amino acids in coil may be due to the algorithms used by this software to identify the position of the amino acids in the secondary structures, since different results were obtained using different predictive software. 

Conversely, other studies that used different approaches identified a set of ten epitopes (IEDB data bank; https://www.iedb.org/home_v3.php; Accessed on 12 December 2022). Only four presented sequences that matched our identified epitopes, while the other six showed no correlation. Due to the possible biological importance of any epitope, an experiment was designed to analyze these peptide sequences using our SPOT synthesis analytical approach (Figure 6). A small library containing all described epitopes was synthesized and reacted with ChVS. Five of the non-correlating epitopes were visualized after a 5 min exposure, which suggests they are minor epitopes based on our selection of major epitopes.

Clinically, the best treatment is prevention through vaccination. However, a potent neutralization effect on a toxin requires a complete blockage though several points or domains of the molecular surface, which cannot be provided by a single antibody. Therefore, our epitope mapping on the planar surface provides superior information describing with greater coverage the set of IgG epitopes responsible for the neutralization of TeNT. The epitopes identified in this study can serve as a basis for constructing a recombinant polyprotein that can replace the use of TT in vaccine preparations since they were recognized for antibodies from human sera. Improving the vaccine would allow the exclusion of the detoxification stage during manufacturing, which causes side effects, and provide a cost reduction and greater efficiency to the process by improving purity and consistency of the antigen [58,59]. 

Tetanus is diagnosed by the patient’s history and clinical signs [57] since there are no universal disposable laboratory tests. Thus, concerning the diagnosis, our ELISA peptide results using the MAP4 epitope peptides revealed that all four epitopes appropriately discriminated between negative and positive samples (*p* < 0.0001). Furthermore, no cross-reactivity was observed, as anticipated from the BLAST analysis criteria. From the ROC analysis, the sensitivity of the in-house ELISA peptides for peptide 215 was 76%, and for the peptides 216–218, 100%. This increase in signal most likely reflects the performance of the selected epitopes. Another benefit of the MAP4-epitope protein design was to increase the specific activity of the molecule to bind antibodies. Using the assay to compare the vaccine coverage of children using the four-epitope peptides, we can conclude that they were useful to investigate the vaccine efficacy over time as the group with older children displayed lower activity, suggesting a need for the application of a booster vaccine.

## 4. Conclusions

Here, we provide new insights into the protection afforded by vaccination against tetanus by describing, at a high resolution, a set of 43 linear B-cell epitopes recognized by human IgG following the administration of a DTP vaccine in children. Three key epitopes that are most likely involved in vaccine protection were identified. Based on its localization, epitope TT-8/G can be directly involved with blocking the enzymatic activity of the neurotoxin, while the epitopes TT-41/G and TT-43/G in TeNT can interfere with neuronal cell receptor binding. Furthermore, the map shows that at least four of the identified epitopes can be used in an ELISA format to assess vaccine coverage. Lastly, a select set of the identified epitopes can serve as a basis for the design of a recombinant multi-epitope vaccine [60], which would avoid the need for a detoxification process and simplify and reduce production costs. 

## 5. Materials and Methods

### 5.1. Human Sera and Ethics Statement 

Ninety-two children aged 1–12 years (median age 7.5 years; group A, 1–4 y, group B, 5–9 y, and group C, 10–13 y) vaccinated with the whole DTP (diphtheria/tetanus/pertussis) with no evidence of acute infection or known history of whooping cough and diphtheria were enrolled in this study. This study also included one hundred sera samples from healthy blood bank donors (HEMORIO). This study was approved by the UNIGRANRIO (CAAE: 24856610.0.0000.5283) study center ethics committee and conducted in accordance with good clinical practice and all applicable regulatory requirements, including the Declaration of Helsinki.

### 5.2. Mapping of Specific B-Cell Epitopes of the TeNT Protein

Seventy-two 15-mer peptides were designed to represent the entire coding region of the 1315-amino-acid sequence of the TeNT protein (P04958) of *C. tetani.* These peptides overlapped by nine residues and were prepared in situ on cellulose membranes automatically according to standard SPOT synthesis protocols [61] using an Auto-Spot Robot ASP-222 (Intavis Bioanalytical Instruments AG, Köln, Germany). Coupling reactions were followed by acetylation with acetic anhydride (4%, *v*/*v*) in N, N-dimethylformamide to render peptides unreactive during the subsequent steps. After acetylation, Fmoc protective groups were removed by the addition of piperidine to render nascent peptides reactive. This same coupling, blocking, and deprotection process added the remaining amino acids until the expected desired peptide was generated. After adding the last amino acid in the peptide, the amino acid side chains were deprotected using a dichloromethane-trifluoracetic acid-triiso-butyl-silane (1:1:0.05, *v*/*v*/*v*) and washed with methanol. Membranes containing the synthetic peptides were probed immediately or stored at −20 °C until use. Negative controls (without peptide), positive controls (IHLVNNESSEVIVHK and GYPKDGN AFNNLDRI from *C. tetani*), and KEVPALTAVETGATN (Piovirus precursor) were included in each assay.

### 5.3. Screening of SPOT membranes

SPOT membranes were washed with TBS (50 mM Tris, 136 mM NaCl and 2 mM KCl, pH 7.4) and then blocked with TBS-T (Tris-buffer saline, 0.05% Tween 20, pH 7.4) containing 1.5% bovine serum albumin (BSA) for 90 min at room temperature. After extensive washing with TBS-T (Tris-buffer saline, 0.05% Tween 20, pH 7.4), membranes were incubated for 12 h with a pool of (*n* = 15) sera from DTP vaccinated children (1:150) in TBS-T + 0.75% BSA. The membrane was washed with TBS-T before incubation with goat anti-human IgG (H+L) labeled with alkaline phosphatase (1:5000; ThermoFisher Scientific Inc., Rockford, IL, USA) in TBS-T + 3% casein for 1h, then washed with TBS-T and CBS (50 mM citrate-buffer saline, pH 7.0). Finally, Chemiluminescent CDP-Star® Substrate (0.25 mM) with Nitro-Block-II™ Enhancer (Applied Biosystems, Waltham, MA, USA) was added to complete the reaction.

### 5.4. Scanning and Measurement of Spot Signal Intensities

Chemiluminescent signals were detected on an Odyssey FC (LI-COR Bioscience, Lincoln, NE, USA) using the same conditions described previously [62] with minor modifications. Briefly, a digital image file was generated at a resolution of 5 MP, and the signal intensities were quantified using the TotalLab TL100 (v2009, Nonlinear Dynamics, USA) software. This program has an automatic grid search for 384 spots but does not offer the automatic identification of possible epitope sequences. Due to this, obtained data were analyzed with two minutes of exposition and the aid of the Microsoft Excel program. (Microsoft, Redmond, WA, USA). An epitope was considered the minor resultant sequence of two or more positive contiguous spots with a signal intensity (SI) greater than or equal to 30% of the highest value obtained from the set of spots on the respective membrane. The signal intensity (SI) used as a background was a set of negative control spotted in each membrane.

### 5.5. Peptides and MAP4 Synthesis

A standard solid-phase synthesis protocol was used to prepare dendrimeric multi-antigen peptides (MAP4) for Peptide 215 (MAP4-VPERYEFGTKPEDFN), 216 (MAP4-EYVPTFDNVIENTTS), 217 (MAP4-EKTLNDYKFQFDSNG) and 218 (MAP4-GTVNTQFQ YEYKIYS) using the Fmoc2-Lys-B-Ala Wang resin (CEM, Corp, Charlotte, NC, USA) as described previously [63]. The same methodology was employed to synthesize single peptides TT-8/G (GGAEELTFTFGGQDGG); TT-41/G (YPKDGNAFNNLDRIL); TT-43/G (GHNGQIGNDPNRDGGG). In this case, two glycines were added at the N- and C-terminus of the TT-8G peptide and one glycine and three glycines at the N- and C-terminus of the TT-42G peptide. Briefly, Fmoc removal was accomplished with 20% piperidine in DMF, and the peptide was released from the resin by trifluoracetic acid treatment in the presence of the appropriate scavengers. Next, the peptide mixture was filtered separately from the resins, washed with 2 × 2 mL TFA, and evaporated in a vacuum at room temperature. Peptides were precipitated with 20 volumes of cold diethyl ether, pelleted, washed with ether (3 × 30 mL), and dried. 

Analysis of each MAP was performed as described previously [64]. For ESI-TOF, the peptides were solubilized in deionized water to a final concentration of 10 µg/mL, and then formic acid was added to a final concentration of 0.1%. The mass spectrometer used was the Water UPLC model Acquity-I Class (Water Corp., Newcastle, Australia), and the samples were injected at 1 µL/min. The range used for ion detection was from 1000 to 11,500 m/z (Figure S2).

### 5.6. Peptide-ELISA Serodiagnosis

The in-house peptide-based ELISA assays were performed as described previously with minor modifications [61]. Briefly, wells of Immulon 4HB flat bottom 96-well microtiter plates were coated with 200 ng of each peptide (215, 216, 217, and 218) in 100 µL of coating buffer (Na_2_CO_3_–NaHCO_3_, pH 9.6) overnight at 4 °C. Next, the plates were washed three times using PBS-T washing buffer (PBS with 0.1% Tween^®^ 20, pH 7.2) and blocked (200 µL) using PBS-T with 2.5% BSA over 1 h at 37 °C. Subsequent, diluted ChVS (100 µL) in coating buffer was applied and the plates were incubated for 2 h at 37 °C. Following several washes with PBS-T, the plates were incubated for 1h at 37 °C with 100 µL of goat anti-human IgG HRP (Sigma, St Louis, MO, USA) diluted in coating buffer (1: 5000), washed, and incubated for 15 min with TMB (3,3′,5, 5′tetramethyl benzidine) as substrate (Sigma, St Louis, MO, USA). Absorbance was measured at 405 nm on a Hidex Sense Microplate Reader, Turku, Finland). The immune response was defined as significantly elevated when optical density was above the defined cut-off calculated as the mean of negative controls multiplied by 3 times the standard deviation. Analysis using the commercial Tetanus IgG-ELISA Kit was conducted as described by the manufacturer (Serion Brazil, Pinhais, Paraná, Brazil). 

### 5.7. ELISA for Specific IgG1, IgG2, and IgG3 Isotypes

The subclass of immunoglobulin induced by some selected IgG epitope antigens (TT-8/G, TT-41/G and TT-43/G) was measured using ELISA, as described above. Anti-human IgG1 (Sigma, St Louis, MO, USA), anti-human IgG2 HRP-labelled antibodies (Bethyl Laboratories, Montgomery, TX, USA), and anti-human IgG_3_ (Sigma, St Louis, MO, USA) were used in 1:5,000 dilutions. For the analysis of IgG3, an additional step was performed using anti-mouse IgG-HRP in a dilution of 1:20,000. Serum samples were tested at a 1:100 dilution. The absorbance was measured at 450 nm, as described above. 

### 5.8. Structural Localization of the IgG Epitopes

The orientation of epitopes in the protein crystallographic structure of the TeNT protein (PDB: 1xdt) was performed using PyMOL (Molecular Graphics System, Version 2.0 Schrödinger, LLC). For the amino acid sequence of epitopes and transmembrane topology visualization, the platform Protter was used (http://wlab.ethz.ch/protter; Accessed on 12 December 2022).

### 5.9. Bioinformatics Tools 

The data bank searches for TeNT were performed on the database UniProtKB (http://www.uniprot.org; Accessed on 3 March 2022) and NCBI. The alignment of the sequences was performed on the T-Coffee server (HTTP://tcoffee.vital-it.ch/cgi-bin/Tcoffee/tcoffeecgi/index.cgi; Accessed on 11 September 2022). The prediction of the secondary structure of the protein was performed by PSIPRED servers (http://bioinf.cs.ucl.ac.uk/psipred; Accessed on 15 November 2022) and CDM (http://gor.bb.iastate.edu/cdm; Accessed on 21 August 2022). Some physicochemical parameters related to the peptides used in this study (molecular weight, theoretical pI, and hydrophobicity) were calculated with the ProtParam (http://web.expasy.org/program; Accessed on 5 August 2022). 

### 5.10. Statistical Analysis

The study population was divided into three age groups: 1–4, 5–9, and 10–13 years. The arithmetic mean titers, standard deviations, and geometric mean titers were calculated using Excel. ELISA tests were analyzed qualitatively and semi-quantitatively. Differences in median serum reactivity between age group sera were compared using the Wilcoxon test on blank corrected OD450 absorbance using R software (version 3.6) and RStudio. Sensitivities and specificities were compared through ROC curves using Graphpad Prism Version 5 (GraphPad Software, San Diego, CA, USA) and SRplot (https://www.bioinformatics.com.cn/srplot (accessed on 12 December 2022)).

## Figures and Tables

**Table 1:** List of the IgG epitopes identified in TeNT through SPOT synthesis and their predicted secondary structure (C, coil; H, helix; S, strand) based on I-TASSER* prediction (https://zhanglab.ccmb.med.umich.edu/I-TASSER; accessed on 28 December 2022). The L-chain is represented by aa 2–457 and the H-chain by aa 458–1315.

Code	Sequence of IgG Epitopes	aa Number	2nd Structure *	Code	Sequence of IgG Epitopes	aa Number	2nd Structure
TT-1/G	DPVNNDTII	10–15	C	TT-23/G	IKTIDNFLEKRYEKW	701–715	H
TT-2/G	VPERYEFGTK	46–55	C	TT-24/G	WLGTVNTQFQ	726–735	H
TT-3/G	IEGASEYYDPNYLRTD	66–80	C	TT-25/G	SGPD	764–767	C
TT-4/G	NRIKNNVAGE	96–105	H+C+H	TT-26/G	INKVFSTPIP	851–860	C
TT-5/G	PYLGSYSLLDKFDTNSNSV	116–135	C	TT-27/G	NLDCWVDNEE	866–875	H+C+H
TT-6/G	EYVPTFDNVIENITS	201–215	C	TT-28/G	INND	889–892	C
TT-7/G	YGMQVSSH	242–250	C	TT-29/G	TSGFNSSVITYPDAQLV	898–914	C+S+C
TT-8/G	AEELFTFGGQD	269–279	C	TT-30/G	GINGKAIHLVNNESE	917–931	C+S+C
TT-9/G	VISCNDPNID	308–317	C	TT-31/G	NDMFNN	944–949	C
TT-10/G	QFDKDSNGQ	331–339	C	TT-32/G	VSASHLEQYGTNEYS	961–975	C
TT-11/G	YNSIMYGFTEIELGK	351–365	H	TT-33/G	SAGEVRQITFRDLPD	1.001–1.021	C+S+C
TT-12/G	LLDDTIYNDTEGFN	389–401	C	TT-34/G	DRLSS	1.039–1.043	C
TT-13/G	MRVNTNAFRN	416–425	S+C+H	TT-35/G	TGLGAIREDNN	1.058–1.068	C
TT-14/G	TNIRENLYNRTASLTDLGGE	446–465	C	TT-36/G	DRCNNNNQYV	1073–1082	C
TT-15/G	EKNSFSEEPFQ	480–490	C	TT-37/G	DFWGNPLRYDTE	1.115–1.126	C+H+C
TT-16/G	YNTKNKPLNFNYSLD	496–510	C	TT-38/G	LKNITD	1.143–1.147	C
TN-17/G	YNLQSKITLPNDRTT	516–530	C	TT-39/G	NAPSYTN	1.153–1.159	C
TT-18/G	EIHNIDDNTIYQY	551–563	C+S+C+S	TT-40/G	YTPNNEIDS	1.180–1.188	C
TT-19/G	TTLQRITMT	571–579	C+S	TT-41/G	YPKDGNAFNNLDRIL	1.211–1.225	S+C+H
TT-20/G	FTNES	623–627	C	TT-42/G	GYNAPGIPLYK	1.228–1.238	C
TT-21/G	FIGALETTGVVLLLE	661–675	H+C+H	TT-43/G	HNGQIGNDPNRD	1.271–1.282	C
TT-22/G	KNLDCWVDNE	865–874	H+C	TT-44/G	TDEGWTND	1.308–1315	C

## Data Availability

The data presented in this study are available on request from the corresponding author.

## Supplementary Materials

The following supporting information can be downloaded at: https://www.mdpi.com/article/10.3390/toxins15040239/s1. Table S1: List of TeNT (P01555) synthetic peptides, and position in the cellulose membrane of SPOT synthesis. The overlapping of positive peptides defined by the epitopes is labeled in red. Figure S1: Mass spectrometry. The peptide 217 (MAP4-EKTLNDYKFQFDSNG) was solubilized in deionized water to a final concentration of 10 µg/mL and then formic acid was added to a final concentration of 0.1%. The mass spectrometer used was the Water UPLC model Acquity-I Class. The samples were electronically injected by the equipment at 1 µL/min. The range used for ion detection was from 1000 to 11,500 m/z. Figure S2: Solvent accessibility area of residues in tetanus toxin using bioinformatics (http://cib.cf.ocha.ac.jp/bitool/ASA/ (accessed on 12 December 2022)).
