# Supplementary material for: High-Throughput IgG Epitope Mapping of Tetanus Neurotoxin: Implications for Immunotherapy and Vaccine Design
Source: Toxins (Basel). 2023 Mar 24;15(4):239. doi: 10.3390/toxins15040239 (PMC10146279; doi:10.3390/toxins15040239)
Supplement: Supplementary file 1 [file toxins-15-00239-s001.zip › toxins-2246173-SI.docx]

**Table S1**. List of TeNT (P01555), synthetic peptides, and position in the cellulose membrane of Spot synthesis. The overlapping of positive peptides defined by the epitopes is labeled in red.


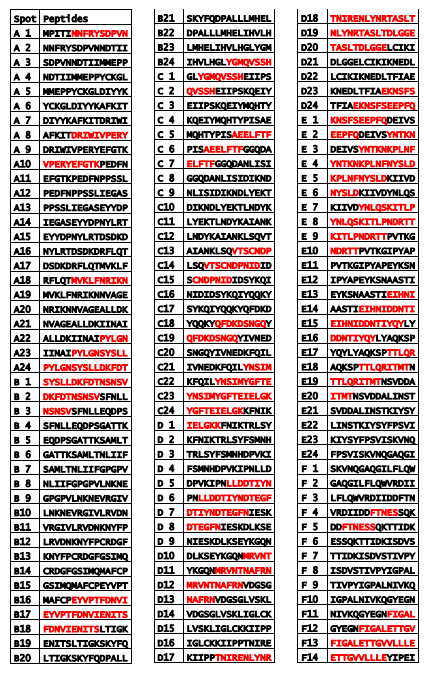


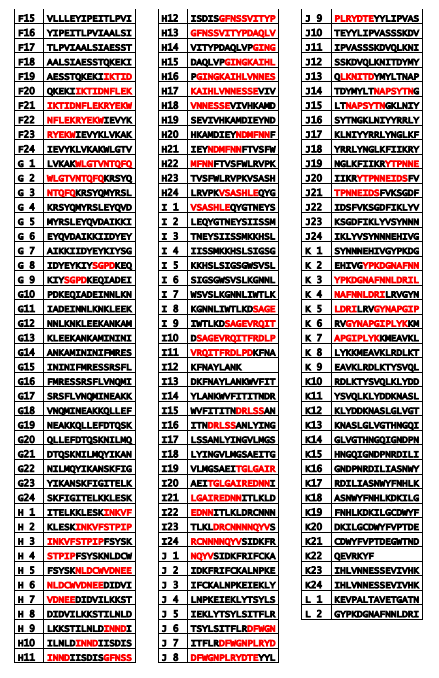


**Figure S1.** Solvent accessibility area of residues in Tetanus toxin using bioinformatics (http://cib.cf.ocha.ac.jp/ bitool/ASA/).


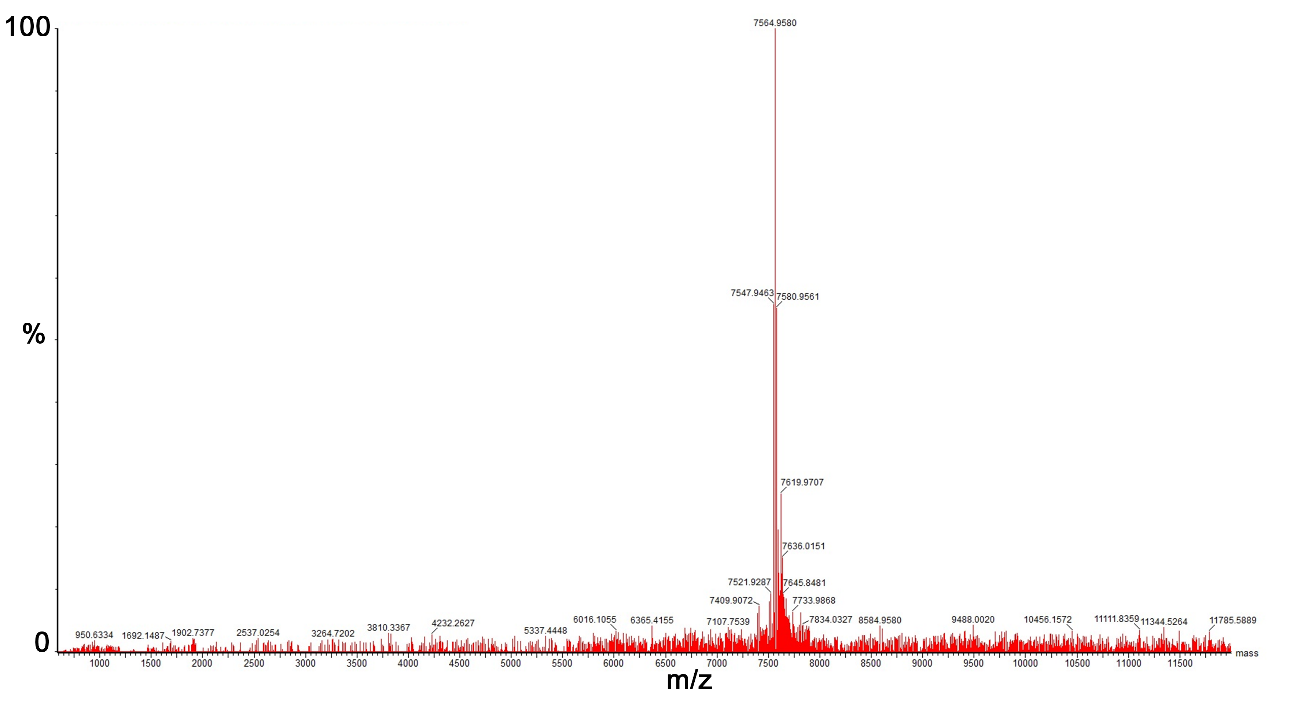


**Figure S2.** Mass spectrometry. The peptide 217 (MAP4-EKTLNDYKFQFDSNG) was solubilized in deionized water to a final concentration of 10 µg/ml and then added formic acid to a final concentration of 0.1%. The mass spectrometer used was the Water UPLC model Acquity-I Class. The samples were electronically injected by the equipment at 1 µl/min. The range used for ion detection ranged from 1000-11,500 m/z.
